# Supplementary figures and images for: Comparative profiling of the synaptic proteome from Alzheimer’s disease patients with focus on the APOE genotype
Source: Acta Neuropathol Commun. 2019 Dec 20;7:214. doi: 10.1186/s40478-019-0847-7 (PMC6925519; doi:10.1186/s40478-019-0847-7)

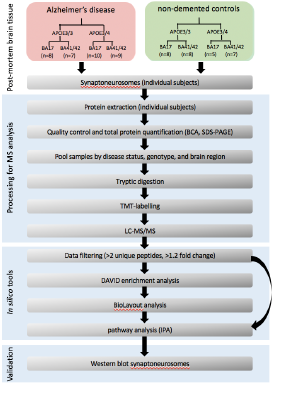

Supplement: Supplementary file 7 — Additional file 7: Figure S1. Proteomics workflow. Samples were prepared from postmortem tissue and processed for proteomics analysis according to the workflow shown. [file 40478_2019_847_MOESM7_ESM.png]

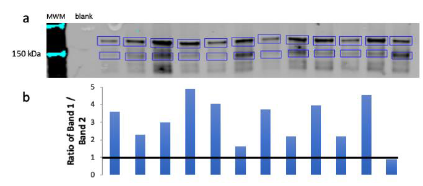

Supplement: Supplementary file 8 — Additional file 8: Figure S2. Protein degradation blot example. Protein degradation blots were completed for all samples using NMDA NR2B antibody (a). The ratio of band 1, which is found in vivo, to band 2, which appears with postmoretem degradation was calculated (b) and samples with a ratio < 1 were excluded from the study. [file 40478_2019_847_MOESM8_ESM.png]

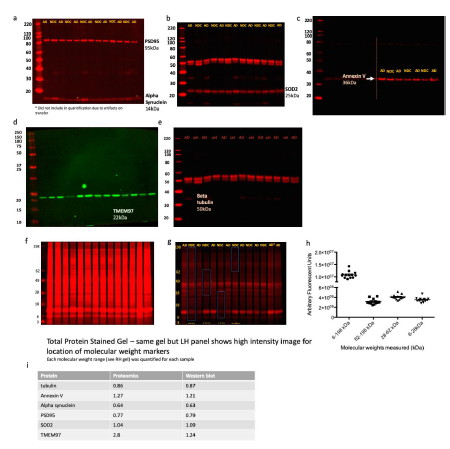

Supplement: Supplementary file 9 — Additional file 9: Figure S3. Validation western blots. Validation western blots (uncropped) of AD vs non demented control (NDC) from BA41/42 of people with APOE3/4 genotype. Full blots are shown for PSD95 and alpha-synuclein (a), SOD2 (b), annexin V (c), TMEM97 (d), beta tubulin (e), and total protein at high intensity (f) and low intensity (g). Each of the molecular weight ranges in g were quantified for each lane, shown in (h). Comparisons between proteomics and western blot data are shown in i. [file 40478_2019_847_MOESM9_ESM.png]
